# Mitochondrial Permeability Transition: A Pore Intertwines Brain Aging and Alzheimer’s Disease

**DOI:** 10.3390/cells10030649

**Published:** 2021-03-15

**Authors:** Kun Jia, Heng Du

**Affiliations:** 1 Department of Pharmacology and Toxicology, The University of Kansas, Lawrence, KS 66045, USA; kunjia@ku.edu; 2 Higuchi Biosciences Center, The University of Kansas, Lawrence, KS 66045, USA

**Keywords:** mitochondrial permeability transition, brain aging, Alzheimer’s disease

## Abstract

Advanced age is the greatest risk factor for aging-related brain disorders including Alzheimer’s disease (AD). However, the detailed mechanisms that mechanistically link aging and AD remain elusive. In recent years, a mitochondrial hypothesis of brain aging and AD has been accentuated. Mitochondrial permeability transition pore (mPTP) is a mitochondrial response to intramitochondrial and intracellular stresses. mPTP overactivation has been implicated in mitochondrial dysfunction in aging and AD brains. This review summarizes the up-to-date progress in the study of mPTP in aging and AD and attempts to establish a link between brain aging and AD from a perspective of mPTP-mediated mitochondrial dysfunction.

## 1. Introduction

The correlation of advancing age with the incidence of Alzheimer’s disease (AD) underpins the argument of AD as a continuum of aging, especially pathological aging [1,2,3]. Recent failures of anti-amyloid beta (Aβ) treatments in clinical trials [4,5,6,7] open up the reappraisal of the importance of aging-related factors in AD pathogenesis. Despite the considerable debate on the relationship between brain aging and AD, the two conditions are proposed, to some extent, to be intrinsically and mechanistically inter-related [3,8,9,10,11,12]. Mitochondrial dysfunction has been repeatedly implicated in brain aging and AD paradigms. Aging and AD brains neuropathologically overlap with each other in oxidative stress, energy deficiency, Ca^2+^ deregulation, as well as alterations in mitochondrial architecture and functional status [9,13,14,15,16,17,18,19]. Analysis of mitochondrial phenotypes in mild cognitive impairment (MCI) that precedes AD further argues for a role of age-associated mitochondrial dysfunction in the development of this disease [20,21,22]. In this context, attempts to conciliate brain aging and AD from a mitochondrial perspective are warranted.

Mitochondrial permeability transition (mPT) through the opening of the mitochondrial permeability transition pore (mPTP) is a mitochondrial response to calcium overloading [23,24] and other cellular stresses such as oxidative stress [25,26], excess inorganic phosphate [27] and depletion of adenine nucleotides [28,29]. mPTP is a nonselective channel that increases the permeability of the inner mitochondrial membrane (IMM) to low molecular-weight solutes and H_2_O. Irreversible mPTP activation results in destructive consequences leading to severe mitochondrial dysfunction [23,30,31,32]. Ever since the characterization of mPT for the first time in 1979 [33], mPTP in neurophysiology and neurological disorders has been under intensive investigation [34,35,36,37,38,39]. With the increased recognition of the involvement of mPTP in brain aging [19,40,41,42,43,44], an mPTP mechanism of mitochondrial dysfunction in age-related disorders such as AD has been highlighted [22,36,40,45,46,47]. In line with the mitochondrial hypothesis of brain aging and AD, these observations query the role of mPTP as an intrinsic link between the two conditions. Therefore, the current review aims to solve the ‘sameness or difference’ debate between mPTP deregulation in aging and AD.

## 2. The Molecular Composition of mPTP

Despite the consensus that mPTP is a hetero-multimer channel contacting the inner mitochondrial membrane (IMM) and outer mitochondrial membrane (OMM), the molecular identity of this mitochondrial pore remains as a conundrum to date. A prevailing opinion was that mPTP is composed of voltage-dependent anion channel (VDAC) and adenine nucleotide translocator (ANT) as the OMM and IMM components, respectively [23]. However, further studies crossed VDAC from the list [48] and demoted ANT from an indispensable component to a less important role, at most, as a mPTP regulator [49,50]. Interestingly, a very recent report of a full inhibition of mPTP in embryonic fibroblasts by genetic depletion of ANT4, the fourth paralogue of ANT, seems to move ANT back to the stage [51]. However, its exclusive abundance in meiotic cells [52] question the role of ANT4 in controlling mPTP in postmitotic cells such as neurons. Aside from these membrane-bound components, cyclophilin D (CypD), a mitochondrial peptidyl-prolyl cis-trans isomerase (PPIase) is a genetically determined key regulator that gates the opening of mPTP [23,53,54], and depletion of CypD remarkably increases mPTP tolerance to calcium loading [23,53,54]. In addition, mitochondrial phosphate carrier (PiC) [55,56] and P53 [57] have also been implicated in the formation and regulation of mPTP.

In recent years, the role of mitochondrial F1Fo ATP synthase in the formation of mPTP has begun to emerge [58,59,60,61]. Mitochondrial F1Fo ATP synthase is formed by three functional entities including the Fo domain integrated in IMM, the F1 domain in the mitochondria matrix, and a peripheral stalk linking the two [62]. The dimerized F1Fo ATP synthase model of mPTP is based on the observation that Ca^2+^ triggers the dimerization of F1Fo ATP synthase to form a high-conductance channel displaying mPTP properties [63,64]. Moreover, Ca^2+^-potentiated F1Fo ATP synthase dimerization is likely in a CypD-dependent manner, probably via the interaction of CypD with oligomycin sensitivity-conferring protein (OSCP), an integrative subunit of F1Fo ATP synthase [64]. Other studies have proposed an F1Fo ATP synthase c subunit-ring model in which the opened channel formed by the c subunits constitutes the IMM component of mPTP [61,65]. The two ATP synthase-centric models of mPTP are reconciled by a two-step process hypothesis, including the dissociation of ATP synthase dimers (step 1), and mPTP opening through the c-subunit ring (step 2) [59]. Although compelling evidence supports F1Fo ATP synthase as the top candidate forming mPTP, Walker et al. cast doubt on this model [66,67]. As the exploration of the molecular structure of mPTP continues, CypD is so far the only well-accepted mPTP regulator.

## 3. The Consequence of mPTP Opening

As a nonselective mitochondrial pore, mPTP has been repeatedly linked to mitochondrial dysfunction. Prolonged opening of mPTP results in mitochondrial depolarization, oxidative phosphorylation (OXPHOS) uncoupling, and reactive oxygen species (ROS) overproduction. In addition, uncontrolled entry of small molecular osmolytes and water into mitochondria through mPTP causes mitochondrial swelling, leading to the disruption of OMM [23,30,31,32]. Moreover, increased mitochondrial permeability favors apoptotic and necrotic cell death through a rapid depletion of ATP [68,69] as well as the leakage of cell death-inducing factors such as cytochrome c and apoptosis inducing factor (AIF) from the inter-membrane space (IMS) [70,71].

Despite the aforementioned deleterious effects of mPTP on mitochondrial function and structural integrity, the physiological function of mPTP is increasingly recognized. Contrary to persistent mPTP-mediated nonselective transportation of small and large molecules, the low-conductance state of mPTP allows a transient diffusion of Ca^2+^ and ROS from mitochondria [72,73]. It is therefore proposed that transient mPTP prevents overloading of mitochondria from Ca^2+^ and ROS accumulation and further contributes to intracellular Ca^2+^ and ROS signaling [73,74,75,76]. Moreover, emerging evidence indicates the relevance of the physiological function of mPTP to neurophysiology. Our very recent study implies a critical role of mPTP in counterbalancing mitochondrial calcium uniporter (MCU)-induced excess calcium accumulation in brain mitochondria [77]. In addition, the physiological mPTP opening is implicated as a critical mitochondrial mechanism for the regulation of neuronal activity-induced filopo-diagenesis and dendritic spine dynamics [35]. The physiological and pathological functions of mPTP seem to raise a “friend or foe” question regarding mPTP’s role in mitochondrial biology. Our speculation is that transient or low-conductance mPTP benefits mitochondrial fitness in physiology and protects mitochondria in stress conditions. However, it may be regarded as certain that in pathological conditions mPTP leads to persistent opening, resulting in severe mitochondrial injury and cellular stress.

## 4. The Induction of mPTP

So far, studies have identified a series of modulators of mPTP opening. These regulatory agents could be further categorized into three groups based on their molecular nature including: (1) ionic regulators such as Ca^2+^, inorganic phosphates (Pi), magnesium ion (Mg^2+^) and proton (H^+^); (2) reactive oxygen species (ROS) such as superoxide, hydrogen peroxide (H_2_O_2_), and hydroxyl radicals; and (3) nucleotides and derivatives of nucleosides including ADP, ATP and NADH. These regulators have distinct impacts on mPTP activation. To be more specific, Ca^2+^, ROS and Pi are activators of mPTP opening, while matrix Mg^2+^, H^+^ and nucleotide derivatives desensitize mPTP [78,79,80,81,82]. Ca^2+^ was historically the first identified mPTP regulator [83], and the interplay between Ca^2+^ and mPTP in regulating mitochondrial homeostasis in health and disease is still under intensive investigation [84]. Although the detailed mechanisms underlying Ca^2+^-mediated mPTP opening have not been fully elucidated, it is generally accepted that the influence of Ca^2+^ on mPTP opening is context dependent. Tenuous matrix Ca^2+^ augmentation triggers transient mPTP activation to alleviate mitochondrial Ca^2+^ overloading and mitochondrial stress; while excess and long-term matrix Ca^2+^ accumulation promotes persistent mPTP formation, resulting in destructive mitochondrial changes [23,35,75,85,86]. Of note, mitochondrial calcium uniporter (MCU) is a determined major pathway for Ca^2+^ entry [87]. In addition, MCU also mediates the entrance of other divalent cations such as Mg^2+^, Sr^2+^, and Mn^2+^ that inhibit Ca^2+^-mediated mPTP opening through competitive inhibition [84]. To this end, mitochondrial Ca^2+^ dynamics and the associated mPTP activation exemplifies, to a certain extent, the interaction between MCU and mPTP.

In addition to Ca^2+^, ROS is another critical regulator of mPTP. A possible mechanism of ROS-mediated mPTP formation is that oxidative modified transmembrane proteins undergo structural misfolding with the assistance of CypD [88,89,90] and form aqueous channels to facilitate mitochondrial membrane permeabilization [91]. A critical and interesting concept related to ROS-mediated mPTP is ROS-induced ROS release (RIRR) [92,93]. In the model of RIRR, when mitochondrial ROS elevates to a “MPT ROS threshold”, mPTP is triggered to release mitochondrial ROS into the cytosol, which further acts on adjacent mitochondria. The RIRR-induced mPT signal amplification sustains even after the removal of the ROS-inducing cell stress events, culminating in the propagation of mPT across all mitochondria. Accompanying mPTP propagation through the RIRR mechanism, mitochondria exhibit Ca^2+^ deregulation and the leakage of Ca^2+^ from affected mitochondria further strengthens the effect of ROS in mediating mPTP activation in neighboring mitochondria, resulting in severe mitochondrial stress and, in a worse scenario, the induction of a mitochondrial pathway for apoptosis [92,93].

## 5. mPTP in Brain Aging

Mitochondrial dysfunction develops with the aging process and constitutes a feature of aging brains [94,95]. Previous studies have repeatedly identified age-related mPTP activation in a variety of tissues. In addition to the liver, the heart, the lymphocytes and others [96,97,98,99,100], our previous and other studies have observed that increased mitochondrial susceptibility to mPTP opening is prominent in brain tissues from aged mice and rats [19,43,96,101,102], indicating a systemic mPTP activation with age. Importantly, such an age phenomenon is determined in human subjects, which is supported by the observations of increased mPTP sensitivity in skeletal muscles from the elderly [103].

mPTP activation leads to mitochondrial ROS production and release, as well as ATP deficiency and loss of metabolites including nicotinamide (NAD) [23,30,31,32]. Therefore, the well-documented Redox stress and metabolic hypotheses of aging [104,105] seem to support a central role of persistent mPTP activation in the aging process. In this context, it would be of importance to understand the mechanisms that promote mitochondrial permeability transition during aging. Ca^2+^ and ROS are strong inducers of mPTP [23]. Ca^2+^ deregulation in neurons develops with age [106] probably through alterations of L type voltage-gated calcium channels [107], Ca^2+^ buffering capacity of endoplasmic reticulum (ER) [108] and glutamate-induced Ca^2+^ influx [109]. Such age-related impairment of Ca^2+^ homeostasis increases the propensity of mPTP opening [110,111], which further exacerbates intraneuronal Ca^2+^ crisis, culminating in a feeding-forward vicious cycle of Ca^2+^-induced mPTP activation during aging. Moreover, increased ROS generation and reduced GSH/GSSG are prominent with age [41,112]. ROS induced mPT is a key process of the mitochondrial free radical theory of aging. In aging cells, mPTP is both an amplifier and an effector of ROS toxicity. mPTP opening disrupts OXPHOS and enhances mitochondrial ROS production. The ROS signal exaggerates through a mPTP dependent RIRR pathway. Inside the mitochondria, mROS causes oxidative damage of mitochondria DNA, mitochondrial proteins, phospholipids and mitochondria dysfunction. Two aging accelerating factors including nuclear DNA damage and NAD+ depletion occur when mitochondrial ROS releases through mPTP and vanquishes the antioxidation system [44]. Moreover, ROS and Ca^2+^ deregulation may synergistically promote mPTP activation. It is proposed that oxidative modification of calcium transporters potentiates excess calcium influx to mitochondria, which subsequently triggers mPTP activation, promoting mitochondrial ROS generation by interfering with mitochondrial electron transfer [44]. In addition to the ROS and Ca^2+^ pathways of mPTP activation, aging cells exhibit changes in mPTP formation-associated proteins. CypD is a crucial regulator of mPTP and increased CypD expression favors mPTP activation [23,113]. Our previous studies as well as other studies have reported increased CypD expression in brain mitochondria in aging paradigms [19,36,45]. Furthermore, along with enhanced mPTP activation, substantial changes of F1Fo ATP synthase including selective loss of OSCP, increased interaction between OSCP and CypD, as well as impaired enzymatic activity and F1FO complex coupling in brain mitochondria from aged mice, were observed in our previous studies [19]. Such an age-related effect on the integrity and function of F1Fo ATP synthase was consistently observed in a P. anserina aging model [114,115] and aged rats [19,116]. Previous studies reported ANT deregulation due to increased oxidative modification in aged Musca domestica [117] and an increase in the ratio of ANT to CypD in aged rat skeletal muscles [118]. However, given the contentious role of ANT in mPTP formation, whether such age-related ANT changes contribute to mPTP activation remains unknown. These observations indicate that mPTP activation constitutes a key mitochondrial change in aging brains and may potentially contribute to the development of brain aging.

## 6. mPTP in AD

In line with the close relationship of aging and AD, the two neurological conditions demonstrate a variety of neuropathological overlaps including oxidative stress, Ca^2+^ deregulation and mitochondrial dysfunction [9,119]. Of note, aging brains and AD share similar changes in proteins associated with mPTP formation. Elevation in CypD expression was reported in postmortem AD brains [36]. Moreover, assays on brain tissues from patients with MCI and AD showed a disease-associated deterioration of OSCP loss [22]. Although the contribution of ANT to mPTP formation is still under debate, mass spectrometry analysis found ANT upregulation in brain tissues from patients with MCI and AD [120]. In view of the inhibitory effect of CypD depletion and OSCP upregulation on mPTP opening in Aβ-rich environments [23,36,40,121], these results support a propensity of mPTP activation in AD. Indeed, increased sensitization of mPTP opening is prominent in brain mitochondria from mouse models with AD-like brain amyloidosis as well as in amyloid-beta (Aβ)-insulted cells [47,122,123]. Despite the appreciation of sameness in the changes of CypD, OSCP and ANT between brain aging and AD, the contribution of Aβ, a key AD mediator to mPTP activation, should be noted. Disturbance of Aβ metabolism including augmented production and impaired degradation leads to excess parenchymal Aβ accumulation [124]. Aβs are peptides produced from sequential cleavages of amyloid precursor protein (APP) [125]. Monomeric Aβ aggregates into more neurotoxic oligomeric forms, the levels of which correlate with the severity of synaptic injury and cognitive impairment in AD [126,127,128,129]. Of note, in addition to extracellular deposition of Aβ, intracellular Aβ accumulation is a feature of AD neurons [130]. Previous studies highlighted the mitochondrial toxicity of intra-mitochondrial Aβ [22,36,131,132,133]. However, the precise pathways for Aβ’s entry into mitochondria remain unresolved so far. The mitochondrial import machinery formed by channel-forming proteins including the translocase of the outer membrane 40 (TOM40) and the translocase of the inner membrane 23 (TIM23) may potentially facilitate mitochondrial uptake of Aβ [134]. Our previous co-immunoprecipitation (coIP) and electro-microscopy studies have determined a physical interaction between mitochondrial Aβ and CypD in AD patients and mouse models of brain amyloidosis [36]. The interplay between Aβ and CypD promotes mPTP activation in brain mitochondria, leading to mitochondrial dysfunction and neuronal stress [36,40]. Moreover, our further examination revealed that OSCP is also a binding partner of Aβ and the formation of Aβ/OSCP complex underlies F1Fo ATP synthase uncoupling and mPTP opening [22,121]. Intriguingly, Aβ is an enhancer of CypD regulation of OSCP by increasing their interaction, strengthening CypD and OSCP-related mPTP formation in Aβ rich milieus [19,121]. Additionally, physical interaction of Aβ with ANT was determined in a previous study [135] and ANT1 deficient mouse showed enhanced resistance to excitotoxic stimulations and Ca^2+^-mediated mPT [136]. Together the impacts of Aβ on neuronal Ca^2+^ deregulation and oxidative stress [137], the interaction of Aβ with mPTP-forming/regulating proteins highlights a unique role of Aβ in promoting mPTP and the engagement of Aβ as a critical modifier of mPTP formation in AD-related pathological setting. In addition to Aβ, aggregation of hyperphosphorylated Tau is another characteristic of AD brains and constitutes a therapeutic target [138]. Modification of Tau protein including hyperphosphorylation and truncation are linked to disease severity and synaptic defects [139]. The interaction with Tau enhances the neurotoxicity of Aβ [140,141]. A potential impact of Tau on mPTP is implicated by the observations of increased mitochondrial ROS in Tau overexpressing mice [142]. Moreover, Tau-mediated mitochondrial membrane potential collapse and oxidative stress are ameliorated by the application of cyslospoin A (CsA), an inhibitor of CypD, in neurons [143]. Tau protein was reported to localize at the OMM as well as within the inner mitochondrial space (IMS) [139,144]. Although the specific mechanisms of mitochondrial uptake of Tau are hitherto undetermined, the presence of mitochondrial Tau implicates that mitochondria are a target of Tauopathy [144]. Neurotoxic amino-terminal (NH2)-derived tau interacts with ANT and ANT/CypD complex in human brain tissues, promoting Aβ/ANT/CypD super-complex [140]. Importantly, genetic depletion of Tau downregulates CypD expression and inhibits mPTP opening [145]. These observations support a connection between Tau and mPTP in AD-related conditions. Therefore, in addition to expression changes of mPTP-related proteins, the involvement of AD mediators including Aβ and Tau may contribute to exaggerated mPTP activation.

## 7. Reconciling mPTP Activation in Brain Aging and AD from a Perspective of Neuronal Stress

**Figure 1:**
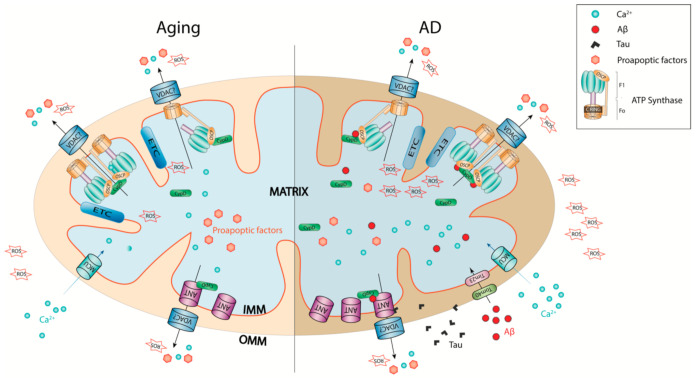
Schematic representation of mPTP in brain aging and AD. mPTP activation is a common mitochondrial pathology that underlies neuronal stress in brain aging and AD. The age-related mitochondrial Ca^2+^ overloading and ROS over-production as well as aberrations of CypD and OSCP are exacerbated by Aβ and Tau in AD-related conditions, leading to increased propensity of mPTP opening. Therefore, mPTP activation may constitute a potential mechanistic link between brain aging and AD and serves as a promising therapeutic target for the treatment of age-related brain disorders including AD. IMM: inner mitochondrial mebrane; OMM: outer mitochondrial membrane; ANT: adenine nucleotide translocator; CypD: cyclophilin D; MCU: mitochondrial calcium uniporter; Tim23: translocase of the inner mitochondrial membrane 23; Tom40: translocase of the outer mitochondrial membrane 40; OSCP: oligomycin sensitivity conferring protein; VDAC: voltage-dependent anion channel.

The sameness pertaining to mPTP activation in aging brains and AD implicates a potential link between the two conditions. First, mitochondrial stress demonstrated by elevated mitochondrial ROS production and calcium deregulation is a common change in aging and AD brains [146]. These mitochondrial abnormalities may promote mPTP activation as well as serve as demonstration in consequence to mPTP formation. Moreover, the expression changes of CypD and OSCP are a mitochondrial response to age-related cellular stress [147,148]. The altered CypD and OSCP expression in the aging brain that overlaps with AD is indicative of similar types of neuronal stress between the two conditions. However, despite the sameness in mPTP activation, the difference in the severity and promoting mechanisms of mPTP formation between brain aging and AD should not be neglected. This difference is closely associated with AD-related Aβ and Tau as confounding factors, which adds a layer of difficulty in arguing for a role of mPTP in the conversion of brain aging to AD (Figure 1). In reference to the mitochondrial cascade hypothesis of AD [149,150,151,152,153,154] and the impact of mitochondrial dysfunction on amyloidosis and Tauopathy [155,156], we therefore hypothesize a model, in which subjects with genetic and/or metabolic risk factors for AD are at high risk of demonstrating mitochondrial stress during aging, leading to mitochondrial ROS and calcium deregulation as well as changes of CypD and OSCP. These changes potentiate mPTP activation and exacerbate mitochondrial stress, contributing to Aβ and Tau abnormalities and the associated mitochondrial and neuronal stress, culminating in a feeding-forward vicious cycle involving in the conversion of brain aging to AD. Of note, cerebral amyloidosis also appears in nondemented older people [157]. Neither significant antemortem cognitive impairment nor neuronal pathology are detected in these subjects. A possible explanation is that there are protective factors which could compensate for Aβ toxicity and neuronal stress that result in Aβ resistance and neuroprotection in these subjects [157]. Future studies of these cases will deepen our understanding of the role of mitochondrial mPTP in the development of AD from aging subjects. Lastly, a paradoxical protection on cell function through mitochondrial dysfunction, known as mito-hormesis, has been recognized in recent years [158]. In this context, aging neurons may utilize the activation of transient mPTP as a strategy to protect mitochondria from excess ROS accumulation and Ca^2+^ overloading. However, such a strategy unlatches persistent mPTP opening with the exacerbation of neuronal stress during aging. In addition, relatively low levels of Aβ are produced in normal aging [159]. Therefore, it is also possible that the bona fide adaption of aging neurons could be hijacked by Aβ, resulting in unrestrained irreversible mPTP opening, culminating in severe mitochondrial dysfunction and the development of AD.

## 8. mPTP as a Therapeutic Target for AD

Given the critical role of pathological mPTP opening in AD pathogenesis, inhibiting mPT could be a promising target for the treatment of AD. Since CypD is the only determined mPTP component, inhibiting CypD could be a practical therapeutic strategy. Indeed, our previous studies and others reported the neuronal protective effecting of inhibiting CypD by either genetic abrogation of CypD or the direct inhibition of CypD by CsA. CypD deficient AD mice and CsA treated mitochondria showed preserved mitochondria membrane potential, enhanced resistance to Aβ-mediated mitochondria swelling, and synaptic function and learning memory [45,160]. However, the immunosuppressive effects and limited permeability through the blood-brain barrier (BBB) of current mPTP inhibitors such as CsA and its derivatives could be a barricade for CypD-targeted therapy [161]. In addition, CypD has its physiological functions including protein folding and chaperon function [76], and manipulation of CypD may also affect mitochondrial protein acetylation [162]. Moreover, CypD-mediated transient mPTP has its importance for neuronal physiology [35]. Therefore, these limitations call for a modulation of CypD activity in a sophisticated manner with caution regarding the physiological functions of CypD and transient mPTP. Another promising target for mPTP regulation is OSCP. One of our recent studies confirmed a protective effect of OSCP restoration against neuronal mPTP activation and synaptic injury in an Aβ-based AD mouse model in vivo [131]. In this scenario, manipulation of OSCP through gene therapy gives hope for a future therapy for AD that needs validation by further clinical trials. Lastly, the molecular identify of mPTP, especially of the pathological form, remains largely unclear. The clarification of mPTP molecular structure and its changes in AD brain will shed light on the development of efficient and safe approaches for mPTP-targeting AD therapy.

## 9. Concluding Remarks

With the increase in the aging population, the incidence of AD is escalating. Despite the familial form of AD, the etiology of sporadic AD remains largely elusive. In recent years, although a mitochondrial cascade theory of AD has been accentuated [154], the detailed mechanisms linking aging and sporadic AD from a mitochondrial perspective are still a topic of intensive investigation. Current studies add credit to a critical role of mPTP activation in mediating mitochondrial dysfunction in aging and AD brains. Although increasing evidence supports the argument that mPTP stands at the nexus of aging and age-related brain disorders, it is still at too early a stage to establish an mPTP-centric hypothesis of the development of sporadic AD. A better understanding of the molecular structure of mPTP, as well as more solid evidence to support mPTP in the conversion of brain aging to AD, are thus called for to strengthen this hypothesis. Nevertheless, in view of the protective effects of mPTP inhibition on mitochondrial function and neuronal activity in aging and AD models [19,36,163], manipulations of mPTP, while preserving its physiological function, seem to be a promising avenue for the treatment and prevention of AD and age-related cognitive decline.
